# Influence of Prior Intense Exercise and Cold Water Immersion in Recovery for Performance and Physiological Response during Subsequent Exercise

**DOI:** 10.3389/fphys.2016.00269

**Published:** 2016-06-28

**Authors:** Peter M. Christensen, Jens Bangsbo

**Affiliations:** ^1^Section of Integrated Physiology, Department of Nutrition, Exercise, and Sports, University of CopenhagenCopenhagen, Denmark; ^2^Team Danmark (Danish Elite Sport Organization)Copenhagen, Denmark

**Keywords:** repeated performance, recovery, cold water immersion, track cycling, peak ⩒O_2_, lactate, potassium, cycling economy

## Abstract

Athletes in intense endurance sports (e.g., 4000-m track cycling) often perform maximally (~4 min) twice a day due to qualifying and finals being placed on the same day. The purpose of the present study was to evaluate repeated performance on the same day in a competitive setting (part A) and the influence from prior intense exercise on subsequent performance and physiological response to moderate and maximal exercise with and without the use of cold water immersion (CWI) in recovery (part B). In part A, performance times during eight World championships for male track cyclists were extracted from the qualifying and final races in 4000-m individual pursuit. In part B, twelve trained cyclists with an average (±SD) ⩒O_2_-peak of 67 ± 5 mL/min/kg performed a protocol mimicking a qualifying race (QUAL) followed 3 h later by a performance test (PT) with each exercise period encompassing intense exercise for ~4 min preceded by an identical warm-up period in both a control setting (CON) and using cold water immersion in recovery (CWI; 15 min at 15°C). Performance was lowered (*P* < 0.001) from qualification to finals (259 ± 3 vs. 261 ± 3 s) for the track cyclists during World championships in part A. In part B, mean power in PT was not different in CWI relative to CON (406 ± 43 vs. 405 ± 38 W). Peak ⩒O_2_ (5.04 ± 0.50 vs. 5.00 ± 0.49 L/min) and blood lactate (13 ± 3 vs. 14 ± 3 mmol/L) did not differ between QUAL and PT and cycling economy and potassium handling was not impaired by prior intense exercise. In conclusion, performance is reduced with repeated maximal exercise in world-class track cyclists during 4000-m individual pursuit lasting ~4 min, however prior intense exercise do not appear to impair peak ⩒O_2_, peak lactate, cycling economy, or potassium handling in trained cyclists and CWI in recovery does not improve subsequent performance.

## Introduction

In sports such as track cycling (4000-m pursuit), cross-country skiing (sprint), and swimming (400-m), athletes compete in a time domain of around 4 min and this typically twice a day due to qualifying races and finals being scheduled on the same day. The influence from such a bout of intense maximal exercise on subsequent performance and the physiological response to exercise is not fully understood.

Regarding performance, one study found that exercise capacity with one-legged isolated knee-extensor exercise was impaired in the second of two time-to-exhaustion tests (~3 min) separated by 60 min of recovery when initiated with normal muscle glycogen levels (~350 mmol/kg dw) but not with an initial high level (~700 mmol/kg dw; Bangsbo et al., 1992). Likewise cycling performance was lowered in the second relative to the first 4–km time-trial (~6 min) when separated by 40 min of recovery including warm-up, with exercise being undertaken at 35°C (Peiffer et al., 2010a). During two 4-min maximal cycling bouts prior maximal exercise was reported to have a possible harmful effect on performance 75 min later with an average reduction in mean power of 4 W (~1%) (Hoon et al., 2014). None of the latter two studies measured VO_2_ and markers of anaerobic metabolism which is warranted to gain insight to what mechanisms are involved in the reduced performance with repeated maximal effort (Peiffer et al., 2010a; Hoon et al., 2014). Other studies report no significant effect of prior maximal exercise on later performance the same day in 2 × 200-m sprint swimming (~2 min) separated by 30 min (Pruscino et al., 2008), 3 × 4-km cycling separated by 17 min (Ansley et al., 2004) and 4 × 1300-m ergometer sprint skiing time (~4 min) separated by 45 min of recovery (Gejl et al., 2016). In the latter study, the 4th interval was faster than the 2nd and 3rd (Gejl et al., 2016) implying that pacing strategies may lower performance in the initial bouts of exercise when a performance task is repeated. Although all studies instructed subjects to perform maximally, the lack of true competition may also have lowered the compliance of the involved subjects and likely the intensity response to the exercise. Thus, it is of interest to compare performance time in real competition in events characterized by multiple races with expected maximal effort lasting around 4 min, to evaluate if performance is reduced when maximal endurance exercise is repeated.

Oxidative metabolism seems to be affected by prior intense exercise. Accordingly, more oxygen was required for ATP resynthesis (lowered P:O ratio) 120 min but not immediately after performing three intense exhaustive cycling bouts (~2–3 min) separated by 5 min of recovery (Tonkonogi et al., 1999). Whole body pulmonary oxygen uptake (⩒O_2_) was not measured, but in another study lower P:O ratio and higher ⩒O_2_ during moderate intensity exercise (reduced exercise economy) was observed 28 h after ultra-endurance exercise (Fernström et al., 2007). With ~4-min of maximal cycling exercise the aerobic contribution has been estimated to be around 85% (Craig and Norton, 2001), thus a lowering of the P:O ratio is likely detrimental to performance. Anaerobic metabolism has also been shown to be affected by prior intense exhaustive exercise since lactate production was lower during the second of two bouts of isolated knee extensor exercise separated by an hour (Bangsbo et al., 1992). During intense exercise reactive oxygen species (ROS) are produced in the contracting fibers. The effect of ROS levels on recovery from intense exercise is complex since factors such as exercise intensity, recovery time and antioxidant status likely all impacts on the net response (Reid, 2008; White and Wells, 2013; Gliemann et al., 2014). Still, ROS has been shown to lower P:O ratio in human mitochondria (Tonkonogi et al., 2000). In turn, ROS appears to reduce calcium sensitivity and likely contributes to development of fatigue (Reid, 2008). ROS also appears to affect potassium handling since the antioxidant n-acetyl cysteine lowered venous potassium accumulation during intense cycling (~6 min) at ~90% ⩒O_2_-max and improved exercise capacity (McKenna et al., 2006). Little is known regarding influence of prior intense exercise on subsequent potassium handling, but increased levels of ROS could be speculated to cause higher potassium accumulation during exercise in the first hour(s) of recovery from intense exercise.

Increased levels of ROS combined with metabolite accumulation such as lactate during intense exercise leads to swelling and inflammation in the period after exercise (White and Wells, 2013). There is evidence to support that reduced afferent feedback induces a more aggressive pacing profile (Amann et al., 2009). Likewise caffeine which lowers perception of effort during intense exercise (Doherty et al., 2004) has been found to improve performance in events lasting 4–6 min such as 2000-m rowing (Christensen et al., 2014) and 1500-m running (Wiles et al., 1992). Consequently interventions that can reduce ROS and inflammation following intense exercise may reduce the proposed detrimental effects on subsequent performance from reductions in exercise economy, impaired potassium, calcium handling, and increased perception of effort.

Cold water immersion (CWI) has been shown to reduce markers of ROS following exercise (Sutkowy et al., 2015) and muscle swelling (Yanagisawa et al., 2003). Numerous studies have deployed CWI to facilitate recovery from exercise with the main outcome being unchanged or improved performance with the proposed mechanism being reduced inflammation (Versey et al., 2013; White and Wells, 2013), however a contributing placebo effect may also be present (Broatch et al., 2014). The majority of these studies centers on resistance type of exercise and running. Little is known about the efficacy of CWI as a recovery strategy for intra-day repeated intense endurance cycling performance. The few studies using cycling exercise tests in the time domain found in intense endurance sports (~1–10 min) have had athletes work in temperature of 35°C (Peiffer et al., 2010a,b), thus the ecologic validity for performance in more temperate conditions is likely low. Interestingly, rating of perceived exertion during constant load cycling was increased ~40 min after a 4-km time-trial in the heat and CWI in recovery could counteract this effect showing that intense cycling can increase perception of effort in subsequent exercise (Peiffer et al., 2010a). Accordingly, it remains to be studied what influence a prior bout of intense endurance exercise has on the subsequent physiological response to exercise in temperate conditions, and if CWI can lower any negative effects from previous intense exercise and improve repeated intense endurance performance in well-trained cyclists.

Thus, the aims of the present study were to (I) evaluate repeated performance on the same day during track cycling World championships in 4000-m individual pursuit (Part A) and (II) evaluate the influence from prior intense exercise on subsequent performance and physiological response to moderate and maximal exercise both with and without the use of CWI in recovery (Part B). It was hypothesized that prior intense exercise would lead to impairments in peak lactate, cycling economy and potassium handling. Moreover, that CWI between intense exercise bouts would reduce these detrimental effects and lower muscle swelling leading to better performance.

## Materials and methods

The study consists of two parts. In part A, performance times during track cycling World championships with expected maximal effort was compared in qualifying and final races performed on the same day in 4000-m individual pursuit lasting ~4 min. In part B, the influence from prior intense exercise on the physiologic response during submaximal- and maximal exercise in the following hours was evaluated together with the efficacy of CWI to facilitate recovery after maximal exercise

### Part A; repeated performance in competition settings

Performance times for male athletes were extracted from the company listed as official time keeper during the World championship in 4000-m individual pursuit in track cycling (http://www.tissottiming.com; October 2015). Both in the qualifying heats and finals two cyclists start at opposite sides on the track. The two races are performed on the same day typically separated by ~3–8 h. The fastest two riders in the 10–14 qualification races, qualified for the final (gold and silver medalists) and the third and fourth fastest riders qualified for the race for third place (bronze medalist).

All available data for the athletes qualifying for the final races was extracted for analysis. Prior to 2007 no data was available. No data for 2007 was obtained since the finale race was stopped before 4000-m due to one rider lapping the competitor. Thus, the analysis was based on performance times during eight World championships (2008–2015).

### Part B; influence from prior intense exercise on subsequent performance and physiological response to moderate and maximal exercise with and without the use of cold water immersion in recovery

#### Subjects

Twelve male road cyclists with an average (± SD) age, height, weight, and ⩒O_2_-peak of 29 ± 6 years, 184 ± 5 cm, 76 ± 9 kg, and 5.07 ± 0.50 L/min or 67 ± 5 mL/min/kg, respectively, took part in the study. Study procedures were approved by the local ethical committee of the capital region of Copenhagen (Region Hovedstaden, H-15003164). All subjects received written and oral information about the study procedures and gave their written informed consent to participate in the study in accordance with the Helsinki declaration.

#### Experimental design

**Figure 1:**
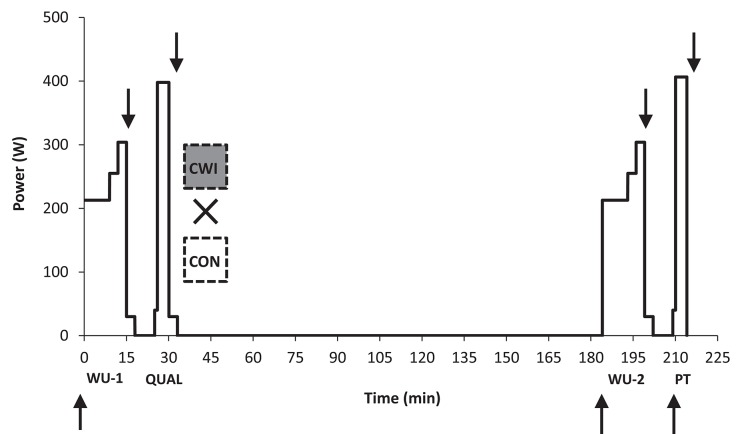
**Study overview**. In a cross-over design trained cyclists performed two identical warm-up periods each lasting 15 min (WU-1 and WU-2) with the former placed before a maximal paced effort replicating a preliminary qualifying race (QUAL) lasting 4:06 min:s on average and the latter placed before an open maximal 4-min performance test (PT) replicating a finale in 4000-m individual pursuit in track cycling. PT was performed 180 min after QUAL in both a control setting (CON) or with the use of cold water immersion (CWI) in the recovery period for 15 min with a temperature of ~15°C. Arrows denote venous blood sampling.

The subjects had 3–4 days with testing during the study. During the first visit an incremental test was carried out to determine the load (gearing) on the ergometer bike used for the performance test on subsequent test days (see below). During the second visit, familiarization to the 4-min maximal performance test (see below) was performed by having the subjects complete the test. Subjects (*n* = 4) with prior experience with the 4-min performance test from our previous study (Christensen and Bangsbo, 2015) did not perform the incremental test, but performed the familiarization trial with same gearing as in the previous study. On the last 2 days, the main experiments took place (Figure 1) using an identical exercise protocol designed to replicate a competition setting with two maximal efforts (a qualifying and final race) each lasting ~4 min separated by 3 h in both a control setting (CON) and with the use of CWI in recovery from the first race. Each maximal effort was preceded by a standardized warm-up (WU), in order to quantify the influence of prior intense exercise (qualifying race) on the physiologic response to exercise. The starting time was identical (± ~60 min) to take into account any influence from the circadian rhythm and half of the subjects started with CWI and the other half with CON.

#### Testing procedures

All testing was performed on a mechanical braked ergometer bike (Monark 839E) controlled by a computer. Subjects were instructed not to perform any training the day before a testing session, maintain their normal training, eating and drinking habits during the study and consume their last meal 3 h prior to meeting in the laboratory and refrain from caffeine intake on testing days. Testing was performed at least 72 h after any bike race. The two main trials were separated by 6 ± 3 days. Pulmonary ⩒O_2_, carbon dioxide excretion (⩒O_2_) and ventilation were measured continuously on-line (Jaeger, Oxycon Pro) during testing and extracted in 5-s intervals for analysis. Before testing both the ergometer bike and the ⩒O_2_ system were calibrated according to the manufactures guidelines.

#### Incremental test

Subjects performed an incremental test (INC) starting at 100 W with increments of 25 W/min until exhaustion. To determine the load on the bike for each cyclist during the performance test (see later) incremental test peak power output (iPPO) was calculated as:

$$\begin{array}{l} \text{iPPO}=\text{last fully completed increment (W)}+\\ \;(\left(\text{s at exhaustive increment}∕60\text{ s}\right)*25\text{W}) \end{array}$$

#### Performance test

A 4-min performance test (PT) mimicking 4000-m individual pursuit in track cycling was used to replicate a finale race in a competition setting with highly trained cyclists typically having a mean power of 360–460 W largely dependent on body weight (Christensen and Bangsbo, 2015). ⩒O_2_-peak (30-s average) obtained during PT has been shown to be similar to values obtained during incremental testing (25 W/min) with values of 5.2 and 5.3 L/min, respectively in highly trained male cyclists (Christensen and Bangsbo, 2015). Load (gearing) was fixed during the test as in track cycling meaning that power output was determined solely by the cycling cadence. The load on the bike was calculated for each cyclist based on the power attained in INC and preferred cycling cadence. The formula used is numeric with the unit being Newton (N)

$$\begin{array}{l} \text{Load PT }(\text{N})=(\text{iPPO/11})+(\text{average cadence last three}\\ \text{ increments INC/}-10)+(10) \end{array}$$

The average load for the cyclists was 40 ± 4 N. During PT, subjects were instructed to complete as much work as possible. Power output was recorded in 1-s intervals for further analysis. The subjects were not informed about the cadence during the test, but received information for every 30-s period completed and for every s in the last 15 s of the test. No verbal support was given during the trials. One min before the start of PT subjects were asked to quantify their “readiness” on a 1–10 scale with 1 being lowest and 10 highest. This was introduced as an indirect measure of muscle swelling. PT was preceded by a 15-min warm-up programme encompassing 9 min at 213 ± 23 W where pulmonary measurements was used to calculate cycling gross efficiency (see later) followed by 3 min at 255 ± 28 W and 3 min at 304 ± 32 W corresponding to intensities eliciting ~65, 75, and 85% ⩒O_2_-max, respectively. Each cyclist choose a cadence on the first experimental day, and it was maintained for each warm-up. PT started 10 min after the warm-up. The first part of this recovery period (0–3 min) was low intensity cycling at 30 W whereas the last part (9–10 min) was cycling at 6 N at ~60 rpm (~40 W) in order to obtain baseline pulmonary measures and synchronize the bike ergometer and ⩒O_2_-system.

#### Main experiments

Each of the two experimental days (CON and CWI) consisted of two periods including an identical warm-up protocol in period 1 (WU-1) and period 2 (WU-2) as described previously followed by a maximal effort mimicking a qualifying race (QUAL) in period 1 and the use of PT mimicking a final in period 2 (Figure 1). Three hours separated the end of QUAL and the start of PT.

QUAL was designed to ensure a reproducible and highly demanding preload to recover from. The total work in QUAL was set as the amount of work performed in PT-familiarization (124 ± 12 kcal) and the load on the bike was similar to the one used during PT. QUAL was performed as a partly paced effort in order to reduce noise on subsequent performance and physiologic measure obtained during exercise. In order to avoid premature fatigue a pacing scheme in QUAL was calculated based on subjects exercising at a mean power of 95% of PT-familiarization for the first 3.5 min. After this preload subjects were instructed to reach the target total work as fast as possible thus requiring maximal effort to recover from as in a competitive qualifying setting. Total time in QUAL-CON and QUAL-CWI was 4:06 ± 0:06 min:s and 4:07 ± 0:05 min:s with the average difference between trials being 1 ± 2 s. The actual pacing by each subject on the first experimental day was used for the second experimental day. Mean power in QUAL was 398 ± 36 in CON and 398 ± 39 W in CWI.

#### Recovery between maximal exercise

After QUAL, active recovery for 3 min at 30 W was performed in both CON and CWI. In CWI, cyclists within 5 min after completing the active recovery, entered cold water (~15°C) to the level of the umbilicus in a seated position with 90° flexed hip and stayed there for 15 min. The water temperature was measured before subjects entered and at the end at the 15-min period being 14.9 ± 1.2 and 14.9 ± 0.5°, respectively. Thereafter passive recovery followed until the start of the second period. In the 20 min period after QUAL cyclists had a standardized energy intake of 1 g carbohydrate and 0.2 g of protein per kg body mass with the use of Chocolate Milk as combined protein and carbohydrate source (Mathilde Cacao, Arla Foods: 3.5 g protein and 9.5 g carbohydrates per 100 ml) and to obtain the remaining carbohydrates soda with negligible caffeine content was served in addition (Faxe Kondi, Unibrew: 10 g carbohydrates per 100 ml, total caffeine content in serving ~20 mg). The second period started 2 h and 35 min after QUAL with WU-2 followed by PT.

#### Blood sampling

Upon arrival at the laboratory and after 10 min of rest a catheter was placed in an antecubital vein for blood sampling. During each of the two experimental days seven venous blood samples (~2 mL) were obtained at rest, after WU-1 and QUAL, before and after WU-2 and PT. Samples were taken 15-s prior to finishing WU-1 and WU-2 due to the rapid recovery of potassium (Nielsen et al., 2004), whilst a 60-s period was used after QUAL and PT. During WU-1 and WU-2 technical difficulties meant that data presented represents 11 subjects. Within 30 min the blood samples were analyzed for lactate and potassium concentration (ABL 720, Radiometer).

#### Pulmonary measurements

In the 15 min warm-up, cycling economy (Gross Efficiency) as in indirect measure of P:O ratio was calculated in the last minute (8–9 min) at the lowest intensity (64 ± 4% ⩒O_2_-max) in WU-1 and WU-2 as the ratio between bike ergometer power output (213 ± 23 W) and whole body energy turnover accounting for RER values (19.6 and 21.1 kJ/L O_2_ for pure fat and carbohydrate metabolism).

⩒O_2_-peak during QUAL and PT was calculated as the highest averaged 30-s value.

### Statistics

Part A: A two-way analysis of variance with repeated measures was conducted with qualifying position (1–4) and race (qualifier and final) as fixed factors and race time as the dependent variable. If a significant main effect or interaction was found a student Newman–Keuls *post-hoc* analysis was performed to identify where the factors differed. Level of significance was *P* < 0.05.

Part B: A paired *t*-test was used to test for differences in mean power during PT between CWI and CON and readiness and blood lactate before PT. A two-way ANOVA for repeated measures was used to evaluate cycling economy, ⩒O_2_-peak, and blood variables using recovery strategy (CWI vs. CON) and either warm-up number (WU-1 vs. WU-2 pre and post or delta change pre and post) or race (QUAL vs. PT) as fixed factors. Likewise, a two-way ANOVA for repeated measures was used to evaluate changes in power output during PT as a function of time using recovery strategy (CWI vs. CON) and pacing (mean power in 30-s intervals) as fixed factors. If a significant main effect or interaction was found a student Newman–Keuls *post-hoc* analysis was performed to identify where the factors differed. Level of significance was *P* < 0.05.

## Results

### Part A

**Figure 2:**
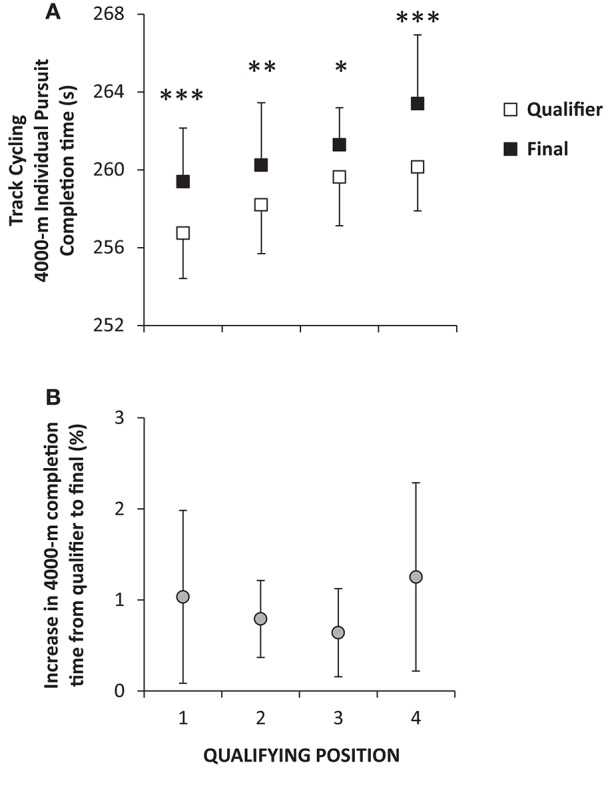
**Average (±SD) completion times during qualifying (open symbols) and final-races (closed symbols) performed on the same day separated by ~3–8 h during eight World championships (2008–2015) for male athletes in 4000-m individual pursuit in track cycling (A) with the relative change in completion time also displayed (B)**. The four best times in qualification proceeded for the finals. ^*^P < 0.05, ^**^P < 0.01, ^***^P < 0.001 significant difference within each qualifying position.

A significant main effect for race (qualifiers vs. finals) was present (*P* < 0.001) with performance being reduced from qualifying heats to finals for all four competitors (*P* < 0.05–0.001). Average time to cover the 4000-m distance for all competitors was 4:18.7 ± 2.6 min:s in qualifiers and 4:21.1 ± 3.2 min:s in the finals amounting to around a 1% reduction in performance (Figure 2). On average 6:28 ± 1:50 h:min separated the two races.

### Part B

#### Performance and maximal exercise response

**Figure 3:**
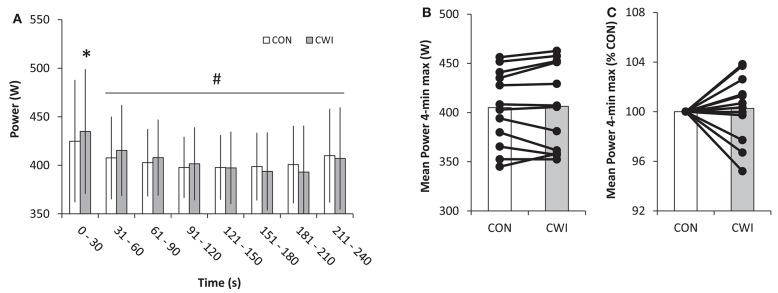
**Power output during a 4-min maximal performance test 3 h after a maximal paced exercise bout lasting ~4 min with normal recovery in between tests as control (CON) or with the use of cold water immersion (CWI) in well trained cyclists**. Mean power (±SD) in 30-s intervals **(A)**, and 4-min mean power in absolute numbers **(B)** and relative to CON **(C)** with individual values shown for the latter two graphs. ^*^P < 0.05 significant difference between CON and CWI. ^#^*P* < 0.05 significant different from 0 to 30 s.

**Figure 4:**
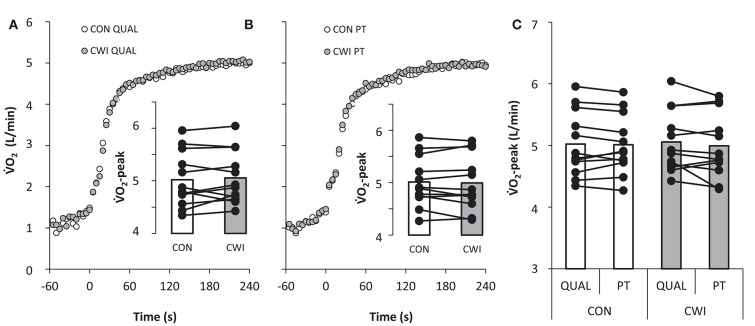
**Pulmonary ⩒O_**2**_ during a maximal paced exercise bout lasting ~4 min mimicking a qualifying race (QUAL; A) and a 4-min maximal performance test (PT; B) mimicking a finale 3 h after QUAL with normal recovery in between tests as control (CON) or with the use of cold water immersion (CWI) in well trained cyclists**. Average ⩒O_2_-peak with individual values is displayed as insert bars **(A,B)** and as a comparison between QUAL and PT **(C)**. For graphical comparison QUAL displays 4 min of exercise but the actual exercise time was 4:06 ± 0:06 min:s in CON and 4:07 ± 0:05 min:s in CWI.

Mean power in PT did not differ (*P* = 0.66) between CWI and CON being 406 ± 43 and 405 ± 38 W, respectively. A main effect was present for pacing (*P* < 0.001) but not recovery (*P* = 0.66) and an interaction (*P* = 0.005) between pacing and recovery was apparent. Mean power in the first 30-s of exercise was 435 ± 64 W in CWI and higher (*P* < 0.05) than 425 ± 63 W in CON with a similar pattern of higher power in CWI than CON from 31 to 60 s (*P* < 0.1; Figure 3). No main effects were present for peak ⩒O_2_ and thus no differences existed for race type (QUAL vs. PT), recovery strategy (CWI vs. CON), or interaction with average values in QUAL and PT of 5.04 ± 0.50 and 5.00 ± 0.49 L/min, respectively (Figure 4). Prior to PT, blood lactate was not different in CWI and CON with respective values of 2.7 ± 0.9 and 2.6 ± 0.8 mmol/L. For peak blood lactate no main effect was observed for race type (*P* = 0.16), recovery strategy (*P* = 0.11) and no interaction was found (*P* = 0.71). Average values for QUAL and PT were 13 ± 3 and 14 ± 3 mmol/L, respectively. Readiness before PT did not differ being 7 ± 1 in CWI and 7 ± 1 in CON.

#### Submaximal exercise response

**Figure 5:**
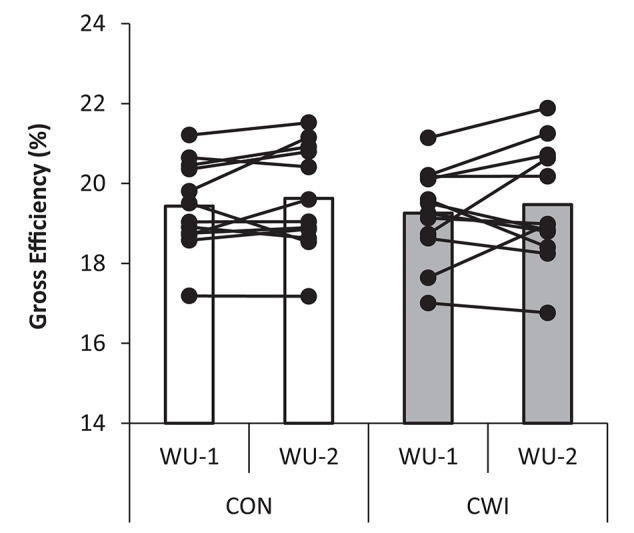
**Gross efficiency with individual values shown during moderate intensity cycling at 213 ± 23 W (~65% ⩒O_**2**_-max) during an identical warm-up protocol performed before (WU-1) and 2:35 h:min after (WU-2) a maximal paced exercise bout lasting ~4 min mimicking a qualifying race with normal recovery in between tests as control (CON) or with the use of cold water immersion (CWI) in well trained cyclists**.

No main effect for warm-up (WU-1 vs. WU-2; *P* = 0.31) or recovery strategy (CWI vs. CON; *P* = 0.50) or interaction (*P* = 0.95) was present for cycling economy being 19.3 ± 1.1 and 19.5 ± 1.5% in CWI and 19.4 ± 1.1 and 19.6 ± 1.3% in CON for WU-1 and WU-2, respectively (Figure 5). In CWI, ⩒O_2_ was 3.22 ± 0.35 and 3.19 ± 0.34 L/min and RER 0.90 ± 0.03 and 0.90 ± 0.04 for WU-1 and WU-2, respectively. In CON, ⩒O_2_ was 3.18 ± 0.40 and 3.17 ± 0.42 L/min and RER 0.93 ± 0.04 and 0.91 ± 0.03 for WU-1 and WU-2, respectively. Cycling cadence in the period used for calculating cycling economy in WU-1 and WU-2 was 96 ± 8 and 98 ± 7 rpm, respectively, in CWI and 97 ± 7 and 96 ± 8 rpm, respectively, in CON. For the entire warm-up period cadence for WU-1 and WU-2 was 94 ± 6 and 95 ± 5 rpm in CWI and 94 ± 5 and 94 ± 5 rpm in CON.

**Figure 6:**
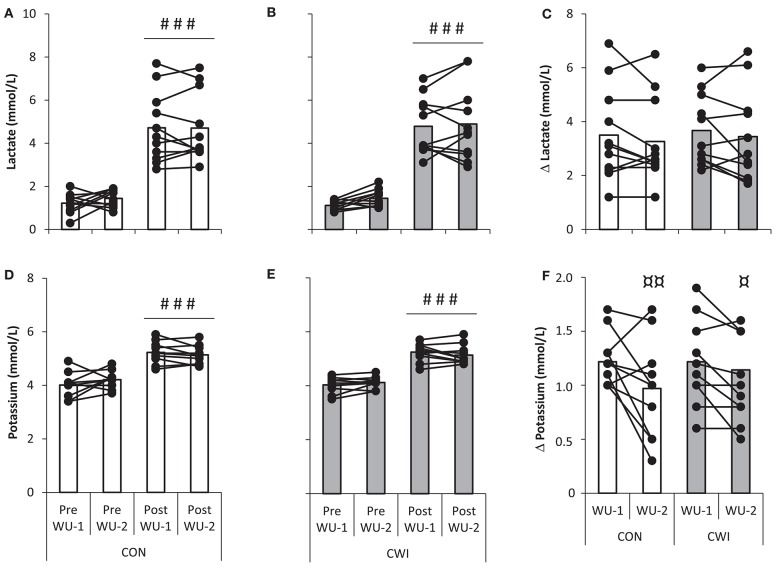
**Venous lactate (top) and potassium (bottom) with individual values shown during an identical warm-up protocol terminating at 304 ± 32 W (~85% ⩒O_**2**_-max) in well trained cyclists**. The warm-up was performed before (WU-1) and 2:35 h:min after (WU-2) a maximal paced exercise bout lasting ~4 min mimicking a qualifying race with normal recovery in between tests as control (CON) or with the use of cold water immersion (CWI). Values pre and post WU-1 and WU-2 in CON **(A,D)** and CWI **(B,E)** and delta change from pre to post for CON and CWI **(C,F)**. ^*###*^*P* < 0.001 significant difference from pre. ^¤¤^*P* < 0.01, ^¤^*P* < 0.05 significant difference from WU-1.

A main effect for warm-up exercise (pre vs. post WU-1 and WU-2; *P* < 0.001) but not recovery strategy or interaction was present for venous lactate being ~1 mmol/L at rest and ~5 mmol/L after WU-1 and WU-2 (Figures 6A,B) with no statistical differences for delta lactate which was similar in CON and CWI (Figure 6C).

A main effect for warm-up exercise (*P* < 0.001) but not recovery strategy or interaction was present for venous potassium being ~4 mmol/L at rest and ~5 mmol/L after WU-1 and WU-2. (Figures 6D,E). A main effect between WU-1 and WU-2 was found for delta potassium (*P* = 0.001), being higher in WU-1 than WU-2 in both CWI (*P* = 0.048) and CON (*P* = 0.006; Figure 6F).

## Discussion

The primary findings in the present study were that performance in the finals was lower than in the qualification performed ~6 h before the finals during the track cycling World championships in 4000-m individual pursuit (~4 min). Moreover, in well trained cyclists peak ⩒O_2_ and blood lactate were the same during intense exercise bouts (~4 min) when separated by 3 h in a design encompassing a simulated qualifying-race (QUAL) followed by a performance test (PT) as a simulated final race. During submaximal exercise, cycling efficiency and blood lactate were not affected, whereas venous potassium increased to a less extent in the period following intense cycling. Lastly, the use of CWI in the initial part of the recovery period after the simulated qualifying race had no impact on subsequent submaximal exercise response and 4-min maximal performance relative to a control setting (CON), but pacing in the first 30-s period was more aggressive following the water immersion intervention.

Part A in the present study showed that the time to cover 4000-m for world-class cyclists was markedly increased (~2.5 s) in the final relative to in qualification. Unfortunately, we do not have access to power data from the races to verify if this lowered performance could be due to other factors than reduced physical capacity, such as changes in temperature or air pressure in the velodromes (Bassett et al., 1999). Changes in air pressure during the day are likely very modest whereas temperature and humidity may have been higher for the finals performed in the evening (in the spring) due to expected higher number of spectators which if anything collectively should have a positive effect on performance. The very best 4000-m individual pursuit track cyclists cover the 250-m laps on the velodromes in around 16-s on average (~8-s separating riders if same speed). Thus, if the finalists did race against very inferior competitors in the qualification-races an aero dynamic advantage would arise when riding in the slipstream, hereby increasing speed for the “same” power compared with in a final in which less difference is expected between competitors. However, this likely had little influence since finalist-cyclists (*n* = 13) who had a time benefit to the competitor in the qualifying race of 5-s or less (0.96 ± 1.72 s) meaning very little slipstream effect still raced slower in finals relative to qualifying (4:20.1 vs. 4:17.8 min:s; *P* < 0.001, paired *t*-test) and that was also the case for the remaining finalists (4:21.2 vs. 4:19.3 min:s; *P* < 0.001) who had a time benefit of 5-s or more (10.7 ± 4.6 s) in the qualifying races. The reduced performance could also be due to more tactical races in the finals with a pacing schedule adjusted to the competitors qualifying time whilst maximal effort always is expected in qualification. Nevertheless, it is considered most likely that the finals are performed at maximal level even if pacing schedules are changed slightly. Hence, performance data from the world championships indicate that in world class track cyclists prior intense exercise leads to decay in the physiologic capacity hours later.

In part B of the present study, the use of CWI after the first intense exercise did not lead to higher performance in the 4 min maximal test 3 h later (Figure 3). A number of studies reviewed in Versey et al. (2013) have reported that CWI similar to the procedure used in the present study can increase recovery from running and resistance exercise. With such type of exercise muscle damage is higher than in cycling due to the high eccentric muscle load. Therefore, it may be that ~4 min of intense cycling and long recovery did not lead to sufficient amount of muscle damage and inflammation for CWI to have a potential to improve recovery. It cannot be, excluded, however, that the previously reported beneficial effects from CWI were due to a placebo effect rather than physiologic changes, since the subjects in a cold water intervention are aware of the intervention. The finding in the present study that the subjects after CWI paced themselves more aggressively in the initial phase of the performance test compared to the control condition, despite their overall “readiness” was similar does support the possibility of a placebo effect. Alternatively, the simple readiness scale before the 4-min performance test was not sensitive enough to detect if perception of effort during exercise—being implicated in regulating pacing (Amann et al., 2009)—was affected by CWI.

The well trained cyclists could maintain their peak ⩒O_2_ (Figure 4) and peak blood lactate when repeating maximal exercise separated by 3 h of recovery implying that cyclists do not acquire a reduced physiologic capacity in the first hours after a maximal bout of exercise. Peak ⩒O_2_ is mainly determined by the muscle mass engaged, cardiac output, blood flow distribution, and ultimately muscle oxygen delivery (Andersen and Saltin, 1985; Mortensen et al., 2005; Romer and Polkey, 2008). Therefore, it does seem that prior intense cycling exercise do not lead to alterations in the cardio vascular system 3 h later, and the observed reduced performance in the world best 4000-m track cyclists likely are caused by other mechanisms. Blood lactate reflect the net balance between production and clearance (van Hall, 2010), implying that muscle lactate production was similar in QUAL and PT with the assumption that lactate clearance was not affected by prior intense exercise. Still, there are limitations in using blood lactate as a reflection of the anaerobic contribution to exercise which is not easily measured (Bangsbo, 1998). Nevertheless, the present finding suggests that the anaerobic metabolism accounting for ~15% of the total energy turnover during a 4 min maximal bout of cycling exercise (Craig and Norton, 2001) is not affected by prior intense exercise when followed by 3 h of recovery, unlike findings with intense isolated knee-extensor exercise using only 1 h of recovery (Bangsbo et al., 1992). However, future studies must include muscle biopsies to establish more clearly if maximal anaerobic metabolism during cycling is reduced several hours after prior intense exhaustive exercise.

The physiological data obtained during standardized warm-up periods using submaximal exercise neither do indicate that prior intense exercise had subsequent detrimental effects. In turn, CWI in recovery did not influence on the physiological response during exercise. Accordingly cycling economy (Figure 5) and blood lactate was the same, and blood potassium accumulation was slightly lower in the latter of two warm-up sessions executed before and after the simulated qualifying race (Figure 6). Previous studies do indicate that intense exhaustive exercise can lead to a lowering of the mitochondrial P:O ratio and higher potassium accumulation which may be caused by increased levels of ROS (Tonkonogi et al., 1999, 2000; McKenna et al., 2006).

Since no detrimental effects were observed for cycling economy and potassium handling, the simulated qualifying race in combination with the recovery period does not appear to have caused increases in ROS or other variables to an extent where it caused physiologic changes as observed previously (Tonkonogi et al., 1999; McKenna et al., 2006). This may be due to the high training status of the subjects (⩒O_2_-peak: 67 ml/min/kg) since training appears to increase the antioxidant system (Gliemann et al., 2014). Alternatively, QUAL may not have been demanding enough although the high values for peak ⩒O_2_ and lactate do indicate that the subjects were maximally taxed. Thus, to explain a potential reduction in physiologic capacity in a time domain of ~4 min following prior exercise of such duration—as indicated by the performance pattern in world-class track cyclists—other mechanisms than reductions in muscle oxygen delivery, cycling economy, and potassium handling are likely involved. One candidate mechanism could be a lowering of muscle glycogen affecting calcium kinetics and fatigue development (Ørtenblad et al., 2013) in the world-class cyclists before the finals relative to before the qualifying race. Accordingly, moderately trained subjects had a glycogen turnover rate of ~70 mmol/kg dw/min during maximal exercise for ~3 min at ~350 W (Iaia et al., 2011). Assuming a higher turnover rate of ~90 mmol/min in the track cyclists qualification races due to an expected work rate of ~500 W for ~4.5 min (Craig and Norton, 2001) this would amount to a reduction in muscle glycogen of ~400 mmol. Adding to this is the glycogen used during each warm-up before the races largely dependent on intensity (2 × ~25 mmol). This net breakdown of ~450 mmol would from a starting level of ~700 mmol (Gejl et al., 2014) and resynthesis from blood glucose and lactate (~50 mmol; Bangsbo et al., 1992) lead to a level of 300 mmol (~60% of initial level) before the finals if no carbohydrate was consumed in recovery. Detrimental effects on calcium release have been observed when glycogen was reduced to ~60–70% of initial levels caused by prior exercise whereas no effect was seen with a reduction of ~40% (Ørtenblad et al., 2011; Gejl et al., 2014). Muscle glycogen can be restored at a hourly rate of ~40–50 mmol with ample carbohydrate intake of ~80 g per h (Ørtenblad et al., 2011; Gejl et al., 2014). Thus, for track cyclists having less than 100 g of carbohydrate in the 4–8 h of recovery, their muscle glycogen levels before the final would likely be around 350 mmol (50% of initial levels) and this may be within a range where it can lower calcium release rate and induce a faster onset of fatigue either in the muscle as a whole or in single fibers low in glycogen (Ørtenblad et al., 2011, 2013; Gejl et al., 2014). Muscle inflammation following qualification can neither be ruled out as a factor if this caused higher afferent input in the final races. Non physiological factors may also be considered such as physiologic pressure and tactical aspects.

To evaluate the influence on recovery by CWI on subsequent performance we chose to have subjects perform a standardized amount of work in QUAL in order to exclude influence from pacing between two maximal trials and potential differences in subsequent fatigue as a result of different power in QUAL. In future studies it will be of relevance to perform two identical performance tests separated by 3–6 h to see if the reduction in performance from qualifier to the finals in world class track cyclists is also present (as a reduction in mean power output), and what mechanisms are responsible. It will also be of interest to obtain and analyze power data in competition for world-class track cyclist in 4000-m pursuit allowing for separation of the physiologic capacity of the riders and the influence from changes in aerodynamic factors and/or race tactics during a competitive day. The present study would have benefitted from additional invasive measures and analysis regarding ROS production and glycogen turnover which may be the scope for future studies concerning intra-day repeated intense exercise in elite athletes.

## Conclusion

The present study showed that during the World championships in 4000-m individual pursuit for male track cyclists the time to cover the distance was longer (~2.5 s) in the final race as compared to qualifying race performed ~6 h earlier indicative of incomplete recovery after the former race. CWI in recovery did not lead to better subsequent performance for a group of trained cyclists in a simulated competitive setting consisting of an identical warm-up protocol before two intense maximal bouts separated by 3 h. Cycling economy, peak ⩒O_2_ and lactate was neither affected by prior intense exercise nor the use of cold water recovery whereas potassium accumulation was slightly lower following intense exercise irrespective of recovery mode.

## Author contributions

PC: Design of study, performing experiments, writing Manuscript. JB: Design of study, writing Manuscript

## Funding

The study was supported by Team Danmark.

### Conflict of interest statement

The authors declare that the research was conducted in the absence of any commercial or financial relationships that could be construed as a potential conflict of interest.
